# Chronic Gastritis

**Published:** 1898-07

**Authors:** Louis A. Kengla

**Affiliations:** San Francisco, Cal.


### CHRONIC GASTRITIS.

BY LOUIS A. KENGLA, M.D., SAN FRANCISCO, CAL.

A report of a very severe case of gastritis was freely copied in medical journals during the year 1896, in which glycozone was successfully used.

At that time, J. W., aged thirty-eight, a blacksmith, came under my care. His illness began in 1894 with the usual symptoms of gastritis. In January, 1895, he had become so much worse that he placed himself in the hands of one of our best physicians, under whose care he continued until November of the same year, when I was consulted.

After hearing his history and the treatment given, I urged him to return to his physician, insisting that nothing more could be done. My protest was in vain.

Examination revealed an emaciated, thin and badly nourished body; his eye, skin and color, fair though pale; his temperature normal; the bowels inclined to constipation with occasional diarrhea with white, pasty, offensive stools; the lungs, heart and kidneys healthy; the liver a trifle small.

There was no painful point and no evidence of enlargement, tumor or ulcer. He was so thin that the abdomen could be most thoroughly examined. His tongue was heavily furred, red at the tip, indented at the edges, and the papillæ red and prominent.

He complained of being unable to take either solid or liquid food even in small quantities without causing heaviness, weight, oppression, pyrosis, eructation of gases, nausea and finally headache and vomiting.

Since 1894 these symptoms had increased in severity; the nausea never ceased, and this whole array of complaints would gradually accumulate in force and energy, overwhelming his system with an attack of headache and intermittent vomiting that would last from three to five days.

In 1895, these storms growing worse, rendered his life almost unbearable. I had been attending him about a week when one of these attacks occurred. He had been vomiting one day before I saw him. The scene was truly pitiable. I found my poor emaciated patient in a small darkened room scarcely able to raise his head, gagging and straining constantly, bringing up finally by the greatest of efforts, a teaspoonful of white glary mucous; his head bound tightly or wrapped in ice cloths; his eyes congested; his cheeks hollow; his skin sallow and pale; his face bespeaking the intense agony he suffered, begging and pleading to those around him for relief from the horrible nausea and retching.

I remained with him an hour, and during that time he was not free for five minutes from efforts at vomiting. His sleepless, aching brain seemed racked to distraction. He would gag, vomit, and fall back exhausted.

This continued three days, gradually lessening. Sleep came only through exhaustion. Every particle of food (liquid or solid) was promptly vomited. During these attacks the temperature was increased from 99 to 103 degrees.

These attacks were always of a similar character, and from November 1, 1895, to July 3, 1896, they occurred every ten days or two weeks.

The physician who had treated him had used drugs, diets, and lavage faithfully and persistently, so that at the outset, I was completely handicapped.

I began with the remedies which had given relief in similar cases, and in turn used acids, alkalies, alteratives, pepsin, digestants, purgatives, tonics, bitters, sedatives, diets, etc., either singly or in combination, until I had exhausted all the resources at my command.

The only perceptible relief came from the use of small doses of diluted hydrochlorid acid between the attacks and a solution of cocaine and morphine during the paroxysm.

About July 3, 1896, I read the article referred to above, and in desperation and despair of ever relieving him, I ordered glycozone one-half, then one drachm, well diluted, twenty minutes before meal-time.

In a few days he said he felt better; within a week he repeated the assertion. To the utter astonishment of myself and his friends, one, two, four and even six weeks passed, without a reoccurrence of his severe symptoms.

About August 20th, he was so much improved, that to hurry matters, I concluded to try lavage again. This was done at 5 P. M, and at 10 that night he was in the throes of an attact, which lasted two days.

He then resumed his glycozone and continued to improve till October 15th, when, on account of inactivity of the bowels and costiveness, he was given two grains of calomel, which brought on a slight headache and considerable nausea.

He had already been taking more food, but from this time it was increased in quantity and character, eating three fairly good meals a day, and enjoying them.

After beginning the use of glycozone, the acid was continued a few weeks, after meals, then left off entirely. No other medicine was used, except occasionally a pill of aloin, belladonna, strychnia, cascara, when bowels were sluggish.

To him glycozone proved the greatest boon, and to me, the relief given was simply wonderful.

It is useless to add, that I have used the remedy in many cases since, and have met with excellent and even astonishing results.

